# History and Description of a Remarkable Monstrosity at Cadiz

**Published:** 1819-08

**Authors:** Franz Xaver Laso

**Affiliations:** Cadiz


FOR THE LONDON MEDICAL AND PHYSICAL JOURNAL.

### *History and Description of a remarkable Monstrosity at Cadiz.*

Communicated by Dr. VON EMBDEN.

*OF the parentage of the monster.*—Antonio Fernandez, a native of Cadiz, 30 years of age, of a sanguineous temperament and healthy constitution, married ten years to Dominica Dadero, of Finala, in the Genoese department, likewise of a sanguineous temper, well made and strong, by trade a cabinet-maker. During the ten years of their matrimonial state, she had brought forth four children,—two boys and two girls. At each of these several deliveries, the artificial delivery of the placenta had been found necessary. During the time of her fifth pregnancy, nothing extraordinary had taken place, nor had her health suffered in the least; so that she may be said to have been completely well all the time. On the 30th of May,

1818, about four o'clock in the morning, she was taken with labour-pains; and by ten the head A, presented itself, in a favourable situation. It had a pretty good and easy passage, as also the trunk, till, when the child had made its progress as far as the abdominal region, some difficulty arose. Two pair of legs now began to make their appearance, in the direction of the head, passing till beyond the navel, presenting themselves at the external pudenda; when Don Joseph Kamino, assisted by the midwife, Donna Antonio Chavo, finally extracted them. This being accomplished, the evolution of the other trunk and head B, took its natural course, without any further obstacle. The placenta was very large, and had but one umbilical chord inserted in its centre: it was however separated entire, and without much difficulty, a few minutes after the children had been delivered.

*External examination.*—The children continued alive from ten o'clock in the morning of the 30th of May, until the 4th of June, thirteen minutes past four o'clock in the afternoon, when the one died three minutes after the other. As long as they were both alive, several changes were observed as well in the respiration as circulation, which were sometimes simultaneous in both, and sometimes varying both in strength and frequency. The voice of child A, was slow and interrupted; but the child B, could neither be brought to cry, nor was its voice ever heard, though its features were plainly expressive of pain. A blackish-blue tinge, which but seldom overspread the whole countenance of the child B, very often coloured that of the other, whose limbs had been convulsed from the very moment of its birth, and whose life seemed in danger from these convulsions. Nevertheless, the natural pulsations of the heart were stronger in this than in the child, B. The intestinal evacuations took place several times, through a single anus, common to both children; yet, twenty hours before their expiration, they became obstructed, and the convulsions of child B increasing, a member of the committee of the Medico-Chirurgical Society, appointed by the stadtholder and governor of this city for the care and preservation of infants, gave them a remedy approved in such cases, viz. syrup of succory and rhubarb. The one, however, was scarcely able to swallow a drop of it; and they already ceased to suck in the night previous to their death. The voluntary motions of both were pretty slow, yet slowest in the convulsed one.

Immediately after their decease, the above-mentioned committee was ordered by the governor to undertake the examination of so extraordinary a phenomenon; which they executed with an attention proportioned to the remarkableness of the case, and noticed the following particulars:

The twins were united by one common abdomen, in the centre of which a single umbilical chord had its origin. The umbilical chord and that region had a greenish appearance, resembling a gangrenous scurf. On one side of the abdomen, the hips, together with the female sexual organs, had their situation: the latter were naturally formed, only that the labia pudenda majora were thick, standing pretty far asunder, and that the usual opening was wanting. The lower extremities belonging to these parts were well shaped, and the buttocks of that side provided with the usual cleft: the opening of the anus was, however, unusually large.

On the opposite side, the iliac region was narrower than the other, but showed not the least mark of sex; in the place of which, a warty excrescence, four lines in length and two in breadth, was seen adhering to the skin, underneath the mons veneris, or the lower belly. The two lower extremities belonging to that side contained, as might indeed be felt, the two bones belonging to both the thighs, but they wanted the patella and the round iliac bone; neither could the tibia and fibula be distinguished. The astragali were deformed, and both extremities covered by a common integument, externally representing but one leg. The feet were joined together till a little beyond the middle of the sole, and five toes made their appearance a little beyond that division: the great toe of the foot belonging to that side standing off at a considerable distance from the next, whereas the other three were connected by an irregular membrane. The hip had, on that side, neither any vestige of buttock nor aperture, but a small groove in lieu of it. The chest, arms, and head, of the child A, were perfectly natural; the chest of child B, a little elevated, and in the epigastric region, about the cardia, a tumor showed itself; on touching which, a fluid could be perceived fluctuating in the pectoral cavity, and beneath it a hard loose substance, which withdrew on being pressed, hiding itself in the right side.

The upper extremities offered nothing remarkable. On the side of the neck, below the maxillary angle, two anomalous tumors, of considerable size and a blackish-blue colour, were met with.

The head of B being rather large, an internal hydrocephalus, or a collection of fluid, was anticipated. The ears had a transverse position, so that their pendulous portions had a straight direction towards the nose. The occiput was flat, and the external bony excrescence in the middle of it so flattened, that it did not hardly appear to exist. Transversely across the occiput, there was a fissure, which, though it seemed to proceed from a fractured bone, penetrated no farther than the skin.

The trunks, posteriorly viewed, exhibited two pair of shoulders, united by one pair of loins, common to both; the spines were plainly distinguishable in the humeral region of either child, but not in the middle of the back.

The whole length, from the farthest extremity of one head to that of the other, measured twenty-one inches. The great transverse diameter, from the points of the toes of the two joined feet to those of the separated ones, measured eighteen inches. The length of the arms, from the points of the fingers of one hand to those of the other, measured sixteen inches. The length of the two joined lower extremities, six inches and two lines; that of the separated ones, seven inches and six lines. The circumference of their common abdomen, thirteen inches; that of the head B, thirteen inches; and that of the head A, thirteen inches and six lines.

Both the children were rather emaciated, and the blackish-blue colour somewhat darker about the shoulders. The limbs, to judge from their motions while alive, were flexible, and the separated lower extremities capable of performing their office; whilst the joined ones only exhibited the motion of contraction and extension, limited to the groins. The laminæ of the head A, were quite soft, but those of B, more ossified.

*Internal examination.*—A semicircular incision having been made in the abdomen, and the viscera having been exposed, a membrane was discovered in the middle, beneath the navel, internally separating the abdomen of the one child from that of the other. This mediastinum consisted of the peritonea of both children, which were united at the bottom; it intercepted all intercourse between the viscera of the two cavities. Between these two membranes lay such parts as were common to both children, and of which it is necessary to speak before we proceed to the examination of the cavities proper to each of them. The membranous mediastinum above-mentioned, had two parietes and two margins, a superior and an inferior one; and two extremities. The paries belonging to the child A, contained a substance of the shape and density of the membrum virile, in which there was a cavity resembling an urethra, but without any external aperture or connexion with the vesica urinaria. The other, belonging to the child B, contained a viscus resembling an uterus, and had on its sides two substances resembling fallopian tubes, terminating in two others not unlike ovaria. All these parts were, however, much larger than those they resembled usually are in new-born infants. The uterus stood in connexion with the anus, or the termination of the cœcum of child B; and the fallopian tubes with the uterus. The superior margin of the membranous septum was joined to the inner paries of the abdomen, and the inferior one to the bodies of the dorsal vertebræ.

One of the two extremities adhered to the os pubis belonging to the hips of B, and the other to that belonging to A. From the body of the dorsal vertebræ to the pubic region of A, passed a ligament; which, passing through the pelvis, joined the inner cavity, and strengthened the adhesion of the common septum.

The vesica urinaria occupied the insterstice left by the laminæ of the above-mentioned septum, towards the thigh of B.

*Proper cavities of child A.*—The condition of the cerebrum, the cerebellum, and the medulla oblongata, was natural; likewise that of the heart and lungs. The stomach was small, and hardly any larger than one of the great guts. The intestines were regular as to situation and size. The liver, spleen, pancreas, and right kidney, were naturally formed; but the left kidney was greatly consumed, and had no ureter; whilst the right ureter opened into the side of the bladder. The farthermost extremity of the cœcum terminated in the anus and the common aperture.

*Proper cavities of child B.*—Between the bones of the head and the membranes, a small quantity of a watery fluid was discovered. The cortical substance of the brain was of an equable red, the medullar substance of a lively colour; the ventricles, and other parts of the cerebrum and cerebellum, and of the spinal marrow, were in their natural state. About half an ounce of coagulated blood was found in the lower occipital ventricles.

On opening the chest, nearly a pound of a bloody fluid flowed from that part where, in the external examination, the tumor had been felt. There was neither any anterior nor posterior mediastinum, no trachea, lungs, or pericardium, to be found; the whole pleura of both sides formed one entire cavity, taken up by the fluid just mentioned, in the middle of which the heart was found floating, which was of a tolerable circumference, and had its situation in the right side. The right side of the heart had a red spotted appearance, and steatomatous excrescences, as big as two peas, were found on the posterior part of it.

The abdomen was rather distended, and filled with a yellowish, thin, purulent, odourless fluid, amounting to about half a pound by weight. The liver wanted the large lobe; and the lesser one had a cordiform shape, with an appendage at its broad side, resting upon the small curvature of the stomach. The gall-bladder was also included in the thickening of the lobe. The stomach was natural in size, and filled with air. The intestines, as far as to the great gut, had their natural situation; the latter, however, wanted the arch, and, instead of the roman S, the gut lay in the right inguinal region, in smooth flexions, passing straight forwards into the cœcum, which terminated in the common anus.

The left kidney was larger than it ought naturally to have been, and its ureter, passing behind the fallopian tube of that side, terminated in the cœcum. The right kidney had its natural size; and its ureter passing behind the fallopian tube of the same side, terminated in that cavity of the child which before we called the uterus.

The pelvis of B was well formed. The upper part of the sacrum formed an articulation with the lateral part of the body of the dorsal vertebræ, and the posterior superior spinous processes of the iliac bone formed the same articulation.

The pelvis of A was smaller, had no sacrum, and had the same articulation.

The spine was an uninterrupted column from the shoulders, and five dorsal vertebræ between the hips, which formed an arch, the concavity of which was directed towards the abdomen, and the convexity towards the loins.

The muscles, arteries, veins, and lymphatics, could not be examined, nor the bones and tumors of the neck; the father of the twins wishing to preserve them for some particular purpose. The number of the ribs could only be observed to be twenty-four.

That the present representation accords with the original paper read before the Medico-Chirurgical Society in the extraordinary meeting of the 8th instant, and which is now kept in the drawer entrusted to my care, is certified by me,

Franz Xaver Laso, *Sec.*

*Cadiz; June* 11*th*, 1818.

